# NAVIGATING COMMUNITY-BASED HOSPICE TRANSITIONS: PALLIATIVE CARE TEAM PROCESSES TO SUPPORT PATIENT-CENTERED CARE

**DOI:** 10.1093/geroni/igae098.2391

**Published:** 2024-12-31

**Authors:** Catherine Mann, Norine Masella, Suzanne Sullivan

**Affiliations:** University at Buffalo, SUNY, Buffalo, New York, United States; University at Buffalo, SUNY, Buffalo, New York, United States; State University of New York Upstate Medical University, Syracuse, New York, United States

## Abstract

Palliative care systems provide a bridge to hospice as goals of care change during chronic, progressive, serious illness management. However, research is lacking on the role of palliative care teams in promoting care transitions outside of the hospital. The aim of this study is to understand the process palliative care teams use to support care transitions in the community to improve earlier acceptance and admission to hospice care by patients and families. Using a Straussian grounded theory approach, we explored processes palliative care teams use to support patient and caregiver transitions in homecare settings. Data were collected through eight recorded and transcribed interviews of palliative care team members (nurses and social workers, n=28) using the constant comparative method. Participants were recruited from three hospice and palliative care agencies serving three counties in New York state. A theory grounded in a process of building trust (e.g., creating safe spaces, listening and learning, building a rapport, meeting patients where they are at), watching and waiting (e.g., deciding if hospice criteria is met, determining readiness for transition, advance care planning), maximizing facilitators (e.g., personalizing care, involving the interdisciplinary team and caregivers, accessing resources), minimizing barriers (e.g., advocating for patient, addressing social determinants of health, navigating regulatory limitations), and getting them over the hill (e.g., educating to reduce stigma, normalizing hospice, reaching the tipping point) was developed. The process identified in the study reveals important mechanistic targets for the development of interventions that promote patient-centered hospice care transitions in the home.

